# Knowledge, willingness, and reported prior prostate cancer testing among men in Palestine: a cross-sectional online survey

**DOI:** 10.3389/fonc.2026.1889495

**Published:** 2026-10-07

**Authors:** Karam Salama, Islam Staiti, Moath Hammad, Said Abu-Hajleh, Ahmed Mousa, Baha Kamel, Ahmad Salah, Osaid Heleseh, Aktham Al-safi, Bashar Al Jaraysa, Bashar Jamal, Momen AbuSaada, Majd Marwan Zaghal, Salahaldeen Deeb, Alhareth M. Amro, Mustafa Nakhala

**Affiliations:** 1 Faculty of Medicine, Al-Quds University, Jerusalem, Palestine; 2 Research Scope, Hebron, Palestine; 3 Faculty of Medicine, Arab American University, Jenin, Palestine; 4 Faculty of Medicine, Palestine Polytechnic University, Hebron, Palestine; 5 Faculty of Medicine, An-Najah National University, Nablus, Palestine

**Keywords:** knowledge, Palestine, prostate cancer, prostate-specific antigen, PSA testing, shared decision-making

## Abstract

**Background:**

Evidence on prostate cancer (PCa) knowledge and PSA-related testing behavior in Palestine remains limited. We assessed general awareness, factual knowledge, willingness to undergo PSA testing if offered, reported prior PSA testing/prostate examination, and factors associated with prior testing.

**Methods:**

We conducted a cross-sectional online survey from 19 November to 21 December 2025 among adult men aged 18 years or older recruited from Palestinian communities. The full cohort comprised 1,047 consenting participants. The exported dataset contained two incompatible response formats for the 12 factual knowledge items; composite knowledge analyses were therefore limited to 568 respondents with complete, unambiguous single Yes/No responses. Knowledge was scored from 0 to 12 using the study answer key. Willingness and prior testing were analyzed as binary outcomes. Multivariable logistic regression assessed factors associated with prior testing.

**Results:**

Mean age was 28.7 ± 12.4 years and 78.6% were aged 18–39 years. Overall, 80.0% had heard of PCa, 26.1% had heard of PSA testing, 31.2% were willing to undergo PSA testing if offered, and 5.6% reported prior PSA testing and/or prostate examination. Among the 568 scorable knowledge respondents, mean knowledge was 7.07 ± 1.84 of 12 and 89.1% had limited knowledge (<80% correct); KR-20 was 0.235. Adequate knowledge was associated with lower willingness (12.9% vs 32.8%; p=0.002) but not prior testing (3.2% vs 6.1%; p=0.526). In the full cohort, older age (aOR 1.08 per year; 95% CI 1.05-1.10), healthcare background (aOR 2.10; 95% CI 1.17-3.77), and first-degree family history (aOR 2.22; 95% CI 1.01-4.86) were independently associated with prior testing. Among never-tested participants, the leading reported barriers were low perceived personal risk (55.3%) and lack of physician/healthcare-provider recommendation (43.1%); multiple responses were allowed.

**Conclusions:**

In this predominantly young online sample, general awareness exceeded detailed factual knowledge, while willingness and reported prior testing were low. The divergence between knowledge and behavior should be interpreted cautiously because knowledge analyses used a restricted subset and the shortened scale had low internal consistency. Findings support culturally appropriate, risk-adapted clinician-patient communication and further validation of locally adapted measurement tools.

## Introduction

Prostate cancer (PCa) is one of the most frequently diagnosed cancers in men worldwide, with incidence rising markedly with age. Established risk factors include older age, family history, and inherited susceptibility, while the contribution of many lifestyle and environmental factors is less certain (1, 2). Early-stage disease is often asymptomatic, making the balance between early detection and avoidance of unnecessary diagnostic and treatment burden an important clinical issue (3).

PSA testing can contribute to earlier detection, but population-wide screening has recognized harms, including false-positive results, biopsy-related complications, overdiagnosis, and overtreatment (4). Contemporary European guidance therefore emphasizes informed, individualized, risk-adapted early detection, with the age at which testing is considered depending on baseline risk and life expectancy rather than routine PSA testing for all adult men (5).

Knowledge and risk perception can influence whether men engage in informed discussions about PCa. A 2025 systematic review of Middle Eastern studies found substantial heterogeneity and generally limited knowledge of screening tools, symptoms, and risk factors (6). Similar knowledge gaps have been reported in Saudi Arabia (7, 8) and in Lebanon (9). The broader cancer burden among men in the Arab world further highlights the need for strengthened cancer prevention, awareness, and earlier detection strategies (10). In addition, a large international cross-sectional study of men aged 40 years or older across 14 Middle Eastern countries found that general PCa awareness was relatively common, whereas awareness of PSA testing and other specific aspects of PCa knowledge remained limited (11). Importantly, knowledge, willingness, and actual testing behavior are distinct constructs and need not move in parallel.

Palestinian evidence remains sparse. A 2025 Al-Quds University master’s thesis among 300 men aged 45 years or older at Beit Jala Hospital reported that 14% had previously undergone screening and identified small correlations among knowledge, attitudes, and practice (12). A broader community-based assessment can complement that older clinical sample. Accordingly, this study aimed to assess PCa awareness and factual knowledge, willingness to undergo PSA testing if offered, reported prior PSA testing/prostate examination, and factors associated with prior testing among adult men recruited from Palestinian communities.

## Methodology

### Study design and setting

A cross-sectional, self-administered online survey was conducted using Google Forms from 19 November to 21 December 2025. Recruitment used non-probability snowball sampling through social-media networks. For analysis, region was categorized as West Bank (including East Jerusalem), Gaza Strip, Palestinian interior, or Other. Because recruitment was non-probabilistic, the study was designed to describe the recruited sample rather than estimate population prevalence.

### Participants and rationale for the age range

The survey targeted men aged 18 years or older. Inclusion of younger adults reflected the broader objectives of assessing health literacy and willingness, rather than an assumption that routine PSA testing is indicated from age 18. Age-specific interpretation was therefore emphasized, and prior testing was examined by age group. Contemporary guidance generally initiates early-detection discussions later in life, with earlier consideration for selected higher-risk men (5). The final dataset contained 1,047 consenting responses from men aged 18 to 79 years, all of whom were included in full-cohort analyses.

The dataset also included a nationality/status item: 935 respondents (89.3%) selected “Palestinian” and 112 (10.7%) selected “Other.” Because the “Other” category was not further specified and legal nationality/status is not equivalent to place of residence, no *post-hoc* nationality-based exclusions were applied. Results are therefore described as pertaining to men recruited in Palestine rather than assuming uniform legal nationality.

### Sample size

No formal *a priori* sample-size calculation was documented in the study records. Recruitment continued throughout the defined data-collection period, and all 1,047 eligible consenting submissions were included in the full analytical cohort.

### Questionnaire source and adaptation

The questionnaire was adapted from a published Lebanese KAP survey by Salah et al. (9), which itself drew questions from earlier publications. The study response spreadsheet documents Arabic wording for sociodemographic variables, PCa/PSA awareness, 12 factual knowledge statements, prior testing, barriers and motivations, and willingness to undergo testing. Several retained factual items closely correspond to items previously used by Morrison et al. (13). The Lebanese instrument contained a longer knowledge scale and a separate multi-item attitude score; therefore, its psychometric properties cannot be assumed to transfer to the shortened Palestinian version. The available study documentation did not contain a formal forward-back translation record, a separate English form, or a distinct Palestinian pilot-validation dataset. Questionnaire fields recoverable from the response spreadsheet are provided in **Supplementary File 1**.

### Knowledge scoring and response-format handling

The 12 factual statements were intended for binary correctness scoring. Each response matching the source-derived answer key was assigned 1 point and an incorrect response 0 points, yielding a 0–12 total. Modified Bloom categories were mapped to attainable integer scores as poor (0-7; <60%), fair (8-9; 60-79%), good (10; 80-89%), and excellent (11-12; ≥90%); poor/fair were grouped as limited (0-9) and good/excellent as adequate (10-12).

The exported dataset contained two response-label formats in the 12 knowledge fields: 653 records used Yes/No labels, 393 used Know/Don’t know labels across all 12 items, and one record contained a mixed pattern. Among the 653 Yes/No-labelled records, 85 contained at least one contradictory dual “Yes, No” selection. Because Know/Don’t know responses cannot establish factual correctness and contradictory dual responses are not scorable, composite knowledge analyses were limited to the 568 respondents with 12 complete, unambiguous single Yes/No responses. The remaining 479 records were retained for all non-knowledge full-cohort analyses. This rule was applied consistently to the knowledge score, knowledge categories, reliability analysis, and knowledge-versus-willingness/testing comparisons (**Figure 1**).

**Figure 1:**
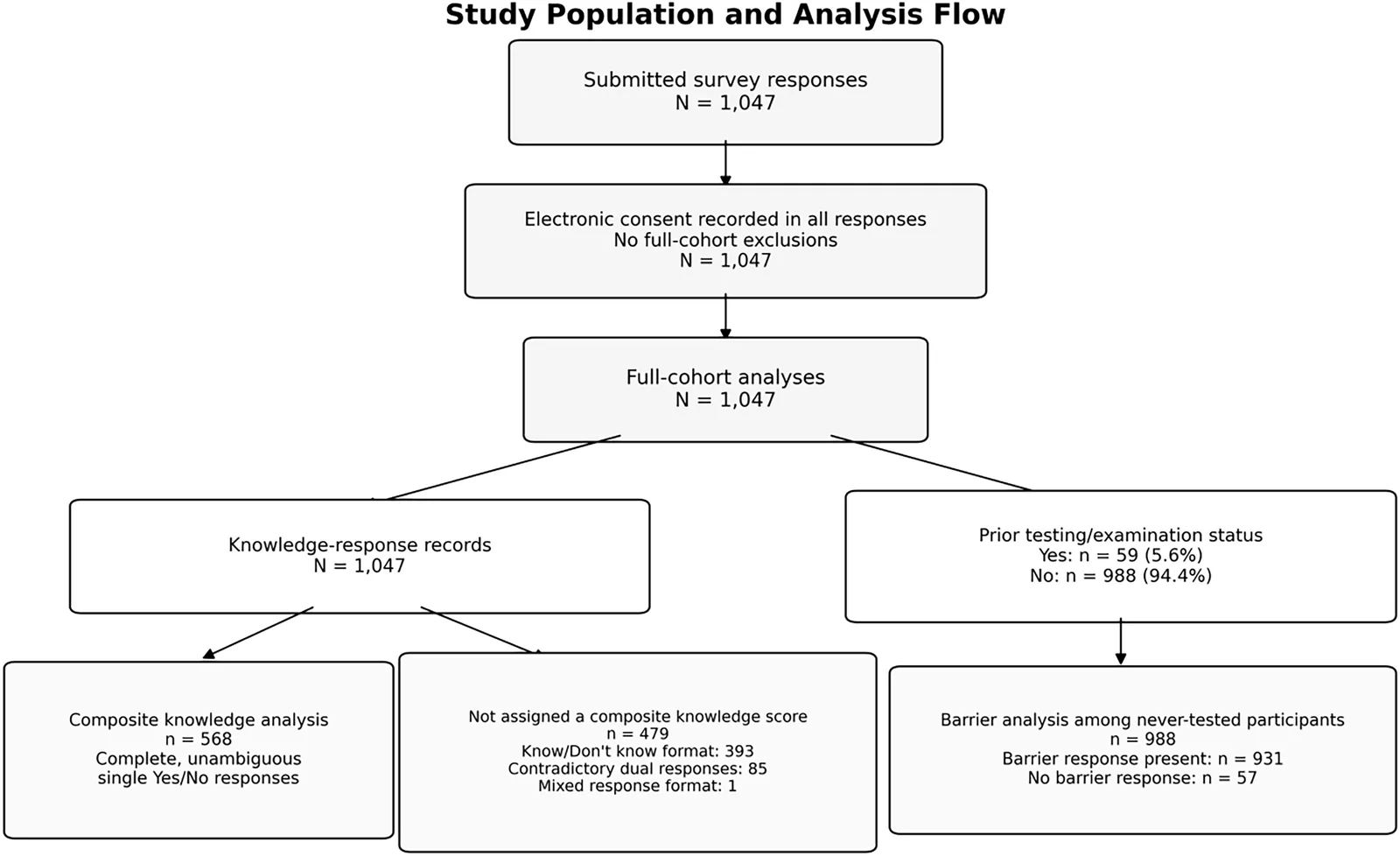
Participant flow and analysis populations. All 1,047 consenting submitted responses were retained in full-cohort analyses. Composite knowledge analyses included 568 respondents with 12 complete, unambiguous single Yes/No responses; 479 respondents were not assigned a composite knowledge score because of incompatible or ambiguous response formats. Among the full cohort, 59 participants reported prior testing/examination and 988 reported no prior testing; 57 of the latter had no recorded barrier response.

### Reliability and outcome definitions

Internal consistency of the 12 dichotomously scored knowledge items was assessed in the 568 scorable respondents using the Kuder-Richardson 20 coefficient (KR-20), which is equivalent to Cronbach’s alpha for dichotomous items. KR-20 was 0.235, indicating low internal consistency. Accordingly, the composite knowledge score was interpreted cautiously and item-level results were also reported. The questionnaire did not contain a multi-item attitude scale comparable to that in the Lebanese source instrument. Willingness to undergo PSA testing if offered was therefore treated as a single binary intention outcome, while practice was represented by self-reported prior PSA testing/prostate examination. These outcomes were not combined into artificial composite attitude or practice scores.

### Missing data and statistical analysis

Core full-cohort variables used for descriptive and regression analyses were complete in the 1,047 submitted records, and no imputation was performed. Conditional reason fields were analyzed only within the relevant subgroup. Among the 988 never-tested respondents, 57 (5.8%) had no recorded reason for not testing. Because barrier questions allowed multiple responses, percentages were calculated using all 988 never-tested participants as the denominator and can sum to more than 100%.

Descriptive statistics are reported as mean ± standard deviation for continuous variables and n (%) for categorical variables. Bivariate associations with prior testing were examined using chi-square tests; Yates continuity correction was used for 2×2 comparisons. To examine cross-domain patterns without creating unsupported continuous attitude or practice scores, knowledge category was compared with willingness and prior testing in the 568 respondents with scorable knowledge data, with phi (φ) reported as an effect size. A full-cohort multivariable logistic regression model evaluated independent associations between prior testing and age (continuous), marital status (married vs other), healthcare background, and first-degree family history. Knowledge was not included in the full-cohort model because 479 participants did not have scorable factual knowledge data; restricting the model to the knowledge subset would have reduced the analysis to 33 outcome events and changed the estimand. Adjusted odds ratios (aORs), 95% confidence intervals (CIs), and two-sided p values are reported; p<0.05 was considered statistically significant. Analyses were performed using IBM SPSS Statistics version 25.

### Ethical considerations

The study protocol was approved by the Institutional Review Board of Al-Quds University. Participants viewed an electronic informed-consent statement before survey completion, and consent was recorded for all 1,047 responses included in the analysis. Data were collected without directly identifying information in the analytical dataset. The study was conducted in accordance with the Declaration of Helsinki.

## Results

### Participant characteristics

All 1,047 submitted consenting records were included in full-cohort analyses. Mean age was 28.7 ± 12.4 years (range 18-79), with 823 (78.6%) aged 18–39 and 224 (21.4%) aged ≥40 years. University-level education (Bachelor’s or higher) was reported by 861 participants (82.2%), and 355 (33.9%) reported a healthcare-related background. Geographically, 715 participants (68.3%) were from the West Bank including East Jerusalem, 273 (26.1%) from the Gaza Strip, 38 (3.6%) from the Palestinian interior, and 21 (2.0%) from other areas. Full characteristics are shown in **Table 1**.

**Table 1:** Sociodemographic and health-related characteristics of the full cohort (N=1,047).

Characteristic	Category	n	%
Age group	18-39 years	823	78.6
	≥40 years	224	21.4
Age, mean ± SD (range)		28.7 ± 12.4 (18-79)	
Marital status	Single	648	61.9
	Married	363	34.7
	Divorced/separated	28	2.7
	Widowed	8	0.8
Educational status	No formal education	31	3.0
	Primary school	12	1.1
	Secondary school	143	13.7
	Bachelor's/university	767	73.3
	Master's or equivalent	71	6.8
	Doctorate or equivalent	23	2.2
Occupation	Student	475	45.4
	Employed	275	26.3
	Self-employed/freelance	216	20.6
	Unemployed	53	5.1
	Retired	28	2.7
Healthcare background	Yes	355	33.9
	No	692	66.1
First-degree family history of PCa	Yes	89	8.5
	No	958	91.5
Smoking status	Never	498	47.6
	Former	103	9.8
	Current	446	42.6
Alcohol use	Never	962	91.9
	Former	50	4.8
	Current	35	3.3
Physical activity	None	348	33.2
	1-2 days/week	366	35.0
	3-4 days/week	189	18.1
	Daily	144	13.8
Residence type	City	478	45.7
	Village	505	48.2
	Refugee camp	47	4.5
	Rural/countryside	17	1.6
Nationality item	Palestinian	935	89.3
	Other	112	10.7
Geographic area	West Bank (incl. East Jerusalem)	715	68.3
	Gaza Strip	273	26.1
	Palestinian interior	38	3.6
	Other	21	2.0

Regional categories used in the analysis were West Bank (including East Jerusalem), Gaza Strip, Palestinian interior, and Other. Percentages may differ slightly because of rounding. PCa, prostate cancer.

### Awareness, knowledge, and cross-domain gap

Overall, 837 men (80.0%) had heard of PCa and 273 (26.1%) had heard of the PSA test. Willingness to undergo PSA testing if offered was reported by 327 (31.2%) participants. Among the 568 respondents with scorable knowledge data, the mean score was 7.07 ± 1.84 of 12 (58.9% correct). Poor, fair, good, and excellent knowledge were observed in 353 (62.1%), 153 (26.9%), 38 (6.7%), and 24 (4.2%) respondents, respectively; 506 (89.1%) therefore had limited knowledge and 62 (10.9%) adequate knowledge (**Table 2**; **Figure 2**). Internal consistency was low (KR-20 = 0.235). Item-level correctness ranged from 32.4% to 79.0% (**Table 3**).

**Table 2:** Awareness, willingness, prior testing, and knowledge indicators.

Analysis set	Indicator				Value	%
Full cohort	Ever heard of prostate cancer				837/1,047	80.0
Full cohort	Ever heard of PSA test				273/1,047	26.1
Full cohort	Willing to undergo PSA testing if offered				327/1,047	31.2
Full cohort	Reported prior PSA testing/prostate examination				59/1,047	5.6
Full cohort	Reported testing/examination in past year				42/1,047	4.0
Knowledge subset	Knowledge score, mean ± SD (0-12)				7.07 ± 1.84	
Knowledge subset	Poor knowledge (0-7)				353/568	62.1
Knowledge subset	Fair knowledge (8-9)				153/568	26.9
Knowledge subset	Good knowledge (10)				38/568	6.7
Knowledge subset	Excellent knowledge (11-12)				24/568	4.2
Knowledge subset	Limited knowledge (0-9)				506/568	89.1
Knowledge subset	Adequate knowledge (10-12)				62/568	10.9
Knowledge subset	KR-20 internal consistency				0.235	
Knowledge-score analyses are limited to 568 respondents with complete, unambiguous single Yes/No responses to all 12 factual items. KR-20 is equivalent to Cronbach’s alpha for dichotomous items.						
Cross-domain knowledge gap within the scorable knowledge subset (n=568)						
Outcome	Knowledge category	n/N	%	χ²*	p	φ
Willing to undergo PSA testing	Adequate knowledge	8/62	12.9	9.38	0.002	-0.129
	Limited knowledge	166/506	32.8			
Reported prior testing/examination	Adequate knowledge	2/62	3.2	0.40	0.526	-0.027
	Limited knowledge	31/506	6.1			

*Yates continuity-corrected chi-square for 2×2 comparisons. Negative φ indicates the outcome was less frequent in the adequate-knowledge group under the displayed coding.

**Figure 2:**
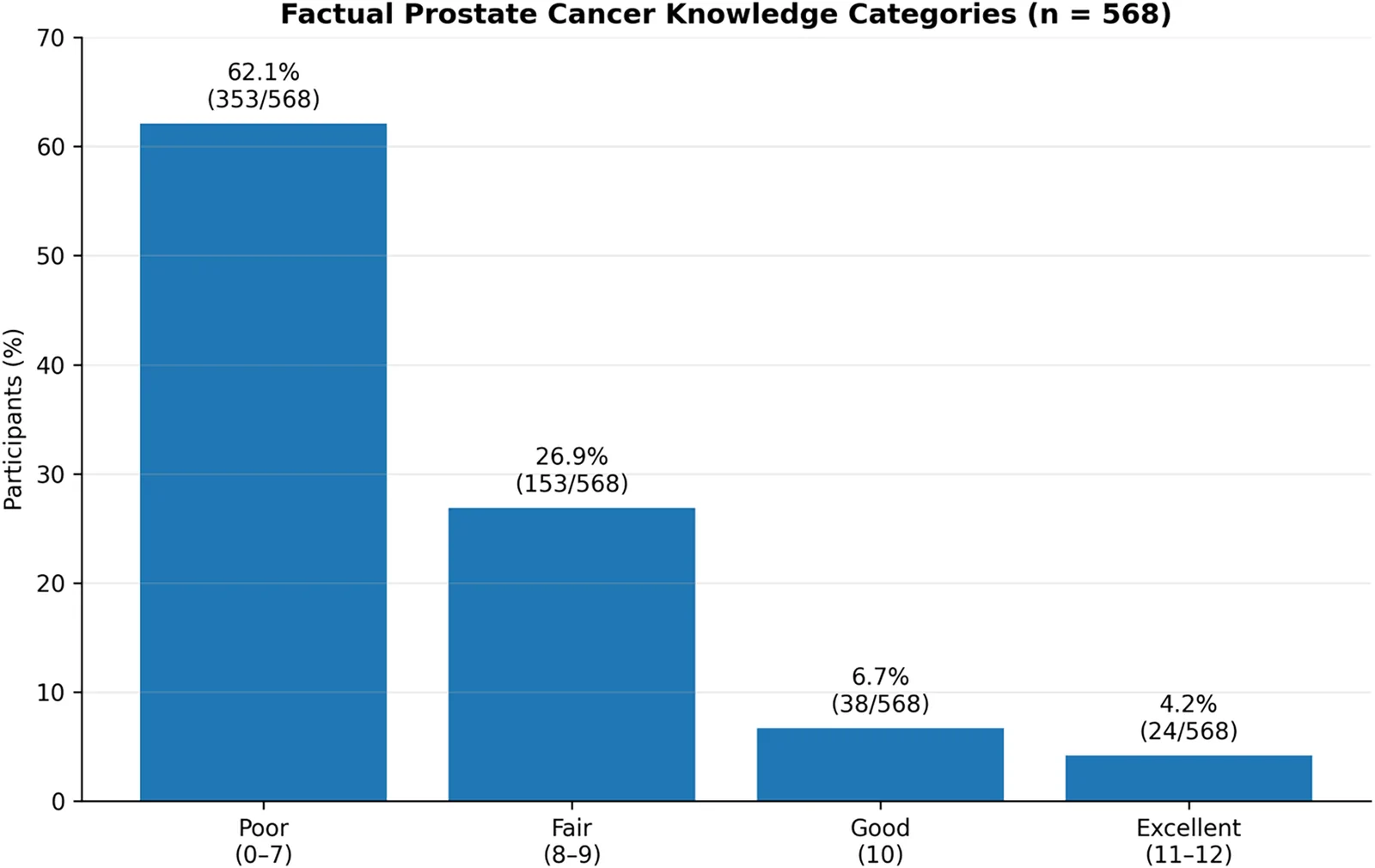
Distribution of factual prostate cancer knowledge categories among scorable respondents (n=568).

**Table 3:** Item-level factual knowledge performance among scorable respondents (n=568).

Item	Statement	Scoring-key answer	Correct n (%)	Incorrect n (%)
K1	Prostate cancer is the most common cancer in men.	True	449 (79.0%)	119 (21.0%)
K2	I should be able to tell immediately if I have a prostate problem.	False	184 (32.4%)	384 (67.6%)
K3	Black men are more likely to develop and die from prostate cancer than other men.	True	187 (32.9%)	381 (67.1%)
K4	Having a family member with prostate cancer increases the likelihood of developing prostate cancer.	True	371 (65.3%)	197 (34.7%)
K5	Frequent nighttime urination may be a sign of prostate cancer.	True	316 (55.6%)	252 (44.4%)
K6	A diet high in fat will reduce the likelihood of developing prostate cancer.	False	379 (66.7%)	189 (33.3%)
K7	The two main tests for detecting prostate cancer are the PSA blood test and digital rectal examination.	True	428 (75.4%)	140 (24.6%)
K8	Conducting only one of the two tests (PSA or DRE) is sufficient to detect prostate cancer.	False	326 (57.4%)	242 (42.6%)
K9	Early screening for prostate cancer cannot determine whether a person has prostate cancer.	False	329 (57.9%)	239 (42.1%)
K10	It is often suggested that Black men over age 40 undergo prostate cancer screening annually.	True	313 (55.1%)	255 (44.9%)
K11	Men aged 50 years and younger are at high risk of developing prostate cancer.	False	292 (51.4%)	276 (48.6%)
K12	A family history of prostate cancer is a risk factor.	True	442 (77.8%)	126 (22.2%)

Correct answers reproduce the source-derived study scoring key used for the analysis. Several statements reflect older or context-dependent screening concepts; the shortened scale was not independently validated in Palestine and had low internal consistency (KR-20=0.235). DRE, digital rectal examination; PSA, prostate-specific antigen.

The cross-domain analysis showed divergence rather than concordance. Among respondents with scorable knowledge, willingness was lower in the adequate-knowledge group than in the limited-knowledge group (8/62 [12.9%] vs 166/506 [32.8%]; χ²=9.38, p=0.002; φ=-0.129). In contrast, prior testing did not differ significantly by knowledge category (2/62 [3.2%] vs 31/506 [6.1%]; χ²=0.40, p=0.526; φ=-0.027). Given the low KR-20 and knowledge-subset restriction, these associations should be treated as exploratory rather than evidence that greater knowledge causes lower willingness.

### Reported prior testing and associated factors

Fifty-nine participants (5.6%) reported ever undergoing PSA testing and/or a prostate examination, and 42 (4.0%) reported testing during the previous year. Prior testing increased with age: 3.0% at ages 18-29, 3.4% at 30-39, 6.8% at 40-49, 25.4% at 50-59, and 32.1% at ≥60 years (**Figure 3**). Men aged ≥40 were more likely to report prior testing than those aged <40 (15.2% vs 3.0%; χ²=46.55, p<0.001). Testing also varied by marital status (global χ²=47.84, p<0.001) and was more common among men with a first-degree family history (11.2% vs 5.1%; χ²=4.64, p=0.031). In unadjusted analysis, healthcare background was not statistically significant (7.0% vs 4.9%; χ²=1.62, p=0.203) (**Table 4**).

**Figure 3:**
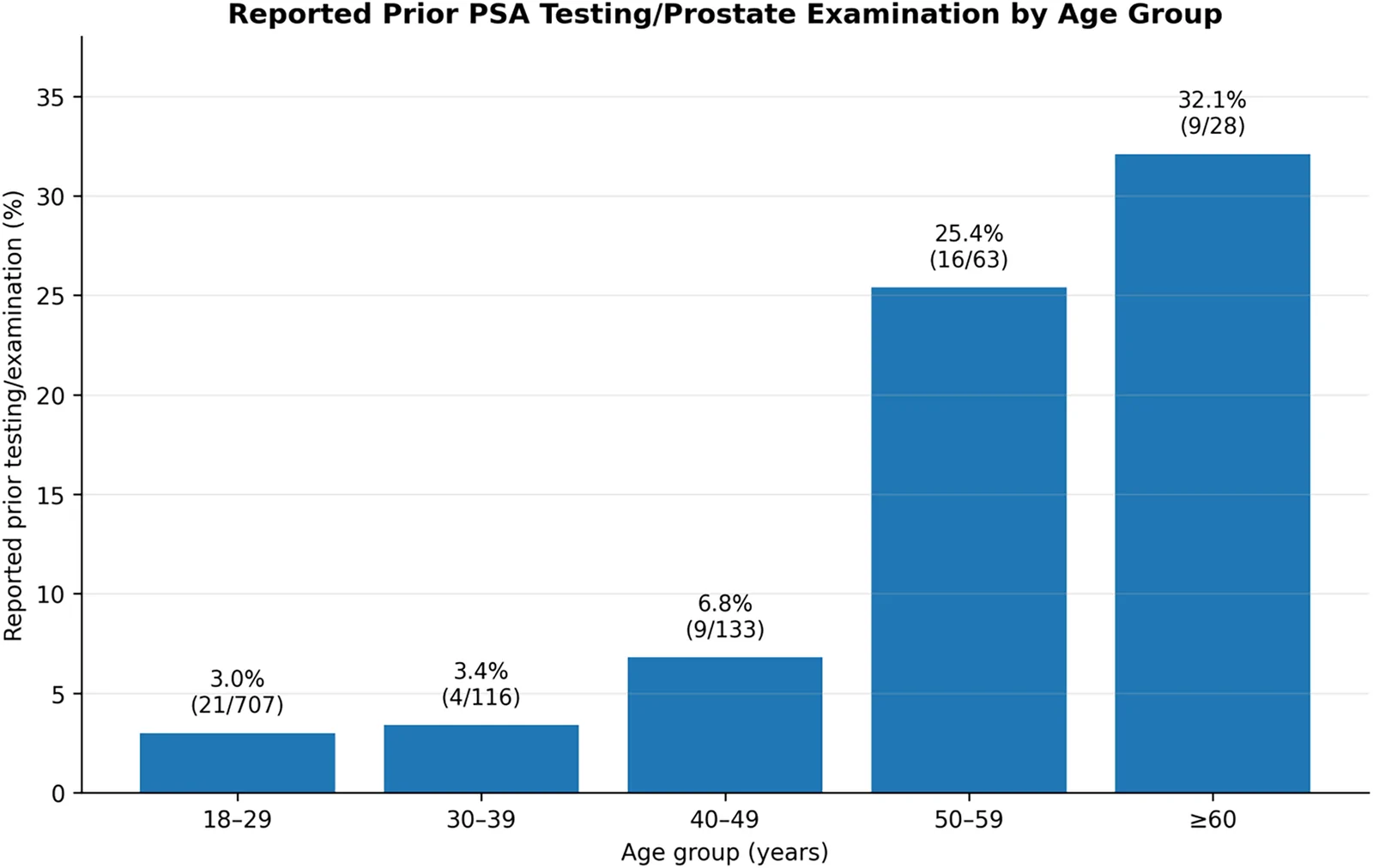
Reported prior PSA testing/prostate examination by age group in the full cohort (N = 1,047).

**Table 4:** Bivariate associations with reported prior PSA testing/prostate examination.

Factor	Category	Tested n/N	%	χ²	p
Age group	18-39 years	25/823	3.0	46.55	<0.001
	≥40 years	34/224	15.2		
Marital status	Single	15/648	2.3	47.84	<0.001
	Married	37/363	10.2		
	Divorced/separated	7/28	25.0		
	Widowed	0/8	0.0		
First-degree family history	No	49/958	5.1	4.64	0.031
	Yes	10/89	11.2		
Healthcare background	No	34/692	4.9	1.62	0.203
	Yes	25/355	7.0		
Knowledge category†	Limited	31/506	6.1	0.40	0.526
	Adequate	2/62	3.2		

2×2 statistics use Yates continuity correction; the marital-status χ² is the global Pearson test across four categories. †Knowledge comparison uses only the scorable subset (n=568); all other comparisons use N=1,047.

In the full-cohort multivariable model (1,047 participants; 59 testing events), age remained independently associated with prior testing (aOR 1.078 per year; 95% CI 1.053-1.104; p<0.001). Healthcare background was also independently associated with prior testing (aOR 2.103; 95% CI 1.174-3.768; p=0.012), as was first-degree family history (aOR 2.220; 95% CI 1.013-4.863; p=0.046). Married status was not independently associated after adjustment (aOR 0.862; 95% CI 0.402-1.849; p=0.702) (**Table 5**).

**Table 5:** Multivariable logistic regression of factors associated with reported prior PSA testing/prostate examination.

Predictor	Adjusted OR	95% CI	p
Age (per year)	1.078	1.053-1.104	<0.001
Married	0.862	0.402-1.849	0.702
Healthcare background	2.103	1.174-3.768	0.012
Family history	2.220	1.013-4.863	0.046

Model: N=1,047, 59 outcome events. Married was coded as married vs all other marital-status categories. Healthcare background and first-degree family history were binary. Knowledge was intentionally excluded because 479 respondents had unscorable factual knowledge responses; a restricted-subset model would have only 33 outcome events.

### Reasons for not undergoing testing

Among the 988 participants who reported never having been tested, the most frequently selected reason was low perceived personal risk (546/988, 55.3%), followed by lack of a physician or healthcare-provider recommendation (426/988, 43.1%), lack of time (124/988, 12.6%), fear of discovering PCa (39/988, 3.9%), and the belief that the test was not useful (32/988, 3.2%). Fifty-seven never-tested participants (5.8%) had no recorded barrier response. Multiple selections were permitted, so percentages exceed 100% when summed (**Figure 4**).

**Figure 4:**
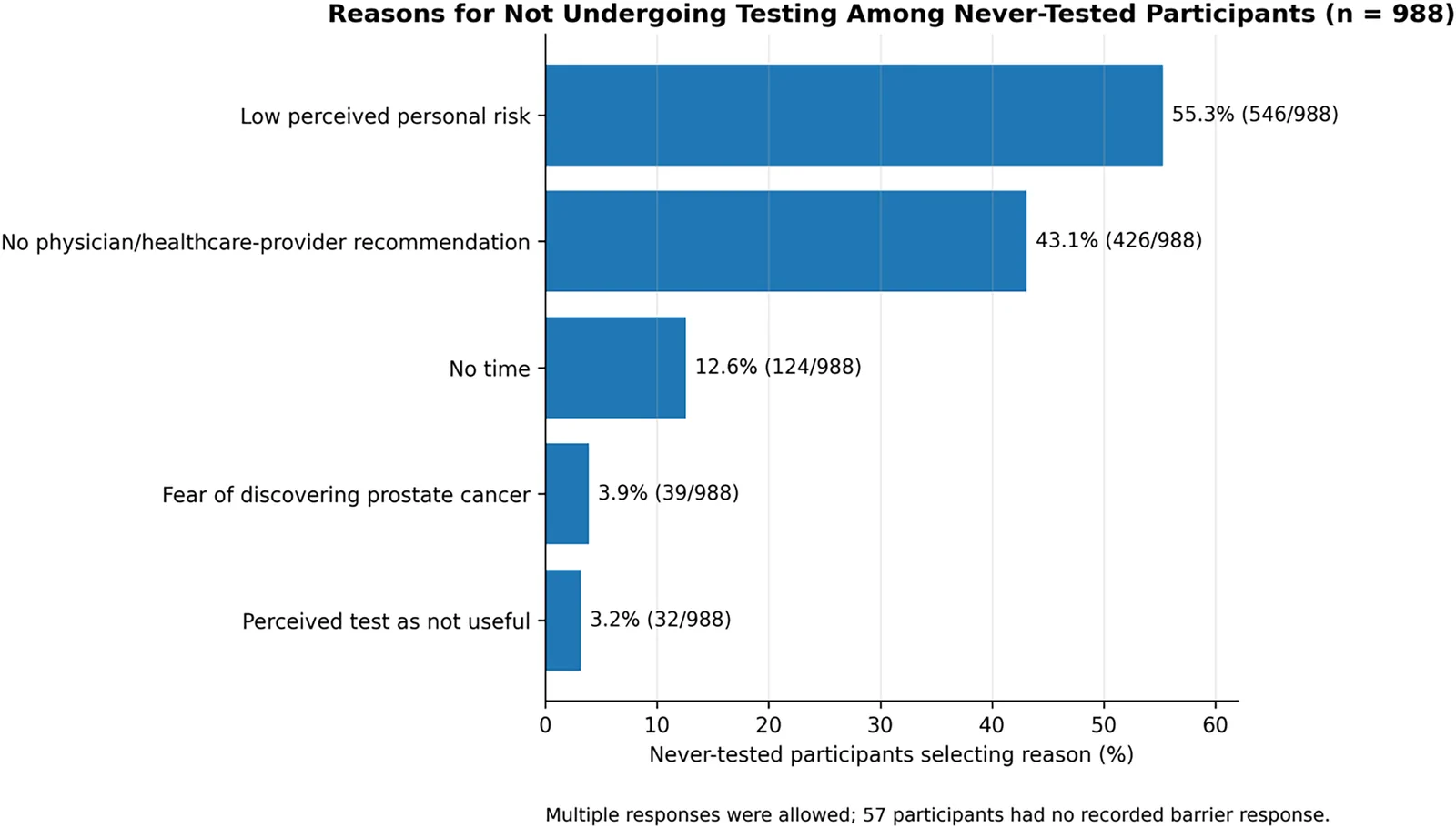
Self-reported reasons for not undergoing testing among never-tested participants (n=988). Multiple responses were allowed; 57 participants had no recorded barrier response.

## Discussion

The findings demonstrate a substantial separation between general PCa awareness, factual knowledge, willingness, and prior testing. Four-fifths of respondents had heard of PCa, yet only 26.1% had heard of PSA testing, 31.2% were willing to undergo testing if offered, and 5.6% reported prior PSA testing/prostate examination. Among the 568 respondents whose knowledge responses could be scored, 89.1% had limited knowledge. These findings indicate that the measured knowledge and behavioral domains did not move in parallel.

The knowledge findings warrant particular caution. The exported dataset contained incompatible response formats in the 12 knowledge items and a small group of contradictory dual responses, so 479 participants could not be assigned a composite factual knowledge score. In addition, KR-20 was 0.235, indicating low internal consistency of the shortened 12-item score in this sample. The Lebanese source questionnaire used a substantially longer knowledge scale and reported higher internal consistency (9); those psychometric properties cannot be transferred automatically to a shortened, contextually adapted version. Accordingly, item-level results are presented alongside the composite score, which should be interpreted as exploratory.

The observed knowledge-willingness divergence was statistically significant but should not be interpreted causally. The adequate-knowledge group was less willing to undergo PSA testing, but the effect was small and the measurement limitations are important. Balanced information about PSA can alter preferences because screening involves trade-offs between possible mortality benefit and harms such as false-positive results, overdiagnosis, and overtreatment (4, 14). Decision-aid trials have likewise shown that informed choice may not always increase testing uptake (15). In the current dataset, however, unmeasured risk perception, age, prior clinician counseling, and the restricted knowledge subset could also explain the pattern.

Age and family history were independently associated with prior testing, consistent with risk-adapted early-detection approaches (2, 5). The adjusted association with healthcare background also remained significant after controlling for age, marital status, and family history. This result differs from the unadjusted comparison and illustrates why multivariable estimates should be reported directly rather than inferred from bivariate percentages. A healthcare background may increase opportunities for health-system contact or knowledge of testing pathways, but the cross-sectional design cannot identify the mechanism.

Low perceived personal risk was the most frequently selected reason for never having undergone testing, followed by lack of physician or healthcare-provider recommendation. This pattern suggests that both personal risk appraisal and clinician communication may influence testing decisions. General-practice research has highlighted the complexity of PSA counseling and clinicians’ role in balancing benefits and harms (16). In the Palestinian context, broader work has also identified system and counseling barriers affecting preventive health behavior (17).

Comparison with prior Palestinian evidence should account for age and setting. Salahat’s 2025 study at Beit Jala Hospital included men aged 45 years or older and reported 14% prior screening (12). The overall 5.6% in the current study is not directly comparable because 78.6% of this online sample was younger than 40 years; among participants aged ≥40, prior testing was 15.2%, closer to the hospital-based estimate. The present findings can also be considered alongside the multinational Middle Eastern study by Sayan et al., in which general awareness of PCa was relatively high but recognition of PSA testing remained limited (11). Although that study included men aged 40 years or older across multiple countries and is therefore not directly comparable with our predominantly younger Palestinian sample, the similar separation between general disease awareness and PSA-specific knowledge is noteworthy. The wider burden of cancer among Arab men further underscores the importance of improving cancer prevention, awareness, and appropriately targeted early-detection strategies across the region (10).

The study has several strengths. It includes a large online sample across multiple Palestinian geographic areas, distinguishes awareness, factual knowledge, willingness, and prior testing rather than forcing them into a single KAP score, and uses an event-efficient full-cohort multivariable model. The analytical reporting also clearly distinguishes the full cohort (N = 1,047) from the subset with scorable knowledge data (n=568), allowing denominators to be followed consistently across the Results, tables, and figures.

Important limitations remain. Snowball sampling and online recruitment overrepresent younger, highly educated, digitally connected men and preclude population-representative prevalence estimates. The study included men far younger than the age at which routine early-detection discussions are usually considered, although this was intentional for the broader knowledge objective. The questionnaire was adapted and shortened without a separately documented Palestinian validation study, and the knowledge scale showed low internal consistency. The response-format inconsistency restricted knowledge analyses to 568 respondents; those respondents were more often university educated than the unscorable subgroup (88.2% vs 75.2%), so selection within the knowledge analysis is possible. Testing was self-reported and the question combined PSA testing/prostate examination without reliably distinguishing screening from symptom-driven diagnostic evaluation. Conditional barrier questions permitted multiple responses and had some missing responses. In addition, the study records did not contain a documented *a priori* sample-size calculation or formal translation/pilot records, limiting the completeness of methodological documentation.

Future research should use a pre-registered protocol, probability-based or mixed-mode recruitment, deliberate oversampling of age groups for whom PSA decisions are clinically relevant, and a prospectively validated Arabic instrument. Questionnaire logic should enforce single mutually exclusive responses for factual items. Prospective studies should distinguish screening from diagnostic testing and evaluate informed choice, risk perception, clinician recommendation, and subsequent testing behavior over time.

## Conclusion

This study assessed PCa awareness, factual knowledge, willingness to undergo PSA testing, and reported prior testing among adult men recruited in Palestine. General awareness was common, but PSA awareness, willingness, and prior testing were low. Among respondents with scorable knowledge data, limited knowledge was frequent, while knowledge did not align consistently with willingness or prior behavior. Older age, healthcare background, and first-degree family history were independently associated with prior testing. Low perceived personal risk and lack of clinician recommendation were the leading reported barriers. Because of non-probability sampling, a young sample, low reliability of the shortened knowledge scale, and a documented knowledge-response format inconsistency, the findings should be interpreted as sample-specific and hypothesis-generating. Future work should validate the instrument prospectively and focus on informed, risk-adapted shared decision-making rather than indiscriminate PSA testing.

## Data Availability

The raw data supporting the conclusions of this article will be made available by the authors, without undue reservation.

## Supplementary material

Supplementary File 1 Questionnaire fields recoverable from the Google Forms response spreadsheet, observed response formats, and analysis scoring key.
