# Supplementary material for: Knowledge, willingness, and reported prior prostate cancer testing among men in Palestine: a cross-sectional online survey
Source: Front Oncol. 2026 Oct 7;16:1889495. doi: 10.3389/fonc.2026.1889495 (PMC13683944; doi:10.3389/fonc.2026.1889495)
Supplement: Supplementary file 1 [file Supplementaryfile1.docx]

**Supplementary File 1**

Questionnaire fields from the Google Forms response spreadsheet, observed response formats, and analysis scoring protocol

Source note: The editable Google Form was not available in the study files used for this analysis. The associated response spreadsheet contained the stored question headers and 1,047 submitted response rows. Arabic wording is reproduced from that response sheet where recoverable. Regional reporting follows the analytic classification used in the study: West Bank (including East Jerusalem), Gaza Strip, Palestinian interior, and Other.

| **Section** | **English description** | **Archived Arabic header** | **Observed response format / note** |
| --- | --- | --- | --- |
| Administrative | Consent to participate | هل توافق على المشاركة ؟ | Yes/No; all 1,047 recorded Yes |
| Administrative | Recruiter/data collector | طُلِبَ مِنكم تعبئة الاستبيان من قِبَل : | Named recruiter |
| Sociodemographic | Governorate/area | 1. المحافظة : | Final analytic region categories used in the manuscript: West Bank (including East Jerusalem); Gaza Strip; Palestinian interior; Other. |
| Sociodemographic | Nationality/status | 2. الجنسية : | Palestinian; Other |
| Sociodemographic | Age | 3. العمر : | Numeric years |
| Sociodemographic | Marital status | 4. الحالة الإجتماعية : | Single; Married; Divorced; Widowed |
| Sociodemographic | Place of residence | 5. مكان الإقامة : | City; Village; Camp; Rural/countryside |
| Sociodemographic | Monthly income | 6. الدخل الشهري : | Low; Middle; High, with explanatory text in Arabic |
| Sociodemographic | Education | 7. الحالة التعليميّة : | No formal education; Primary; Secondary; University; Master or equivalent; Doctorate or equivalent |
| Sociodemographic | Occupation | 8. المهنة : | Student; Employed; Self-employed; Unemployed; Retired |
| Sociodemographic | Healthcare background | 9. هل تعمل في مجال الرعاية الصحية / أو لديك خلفية في الرعاية الصحية؟ | Yes/No |
| Sociodemographic | First-degree family history | 10. تاريخ عائلي لسرطان البروستات (اقارب من الدرجة الأولى: الاب، الاخ، الجدّ، الابن): | Yes/No |
| Sociodemographic | Smoking, including waterpipe | 11. التدخين (يشمل الأرجيلة): | Never; Former; Current |
| Sociodemographic | Alcohol use | 12. شرب الكحول : | Never; Former; Current |
| Sociodemographic | Physical activity | 13. النشاط البدني : | None; 1-2 days/week; 3-4 days/week; Daily |
| Awareness | Ever heard of prostate cancer | هل سمعت من قبل عن سرطان البروستات؟ | Yes/No |
| Awareness | Ever heard of PSA test | هل سمعت من قبل عن فحص المستضد النوعي للبروستات (PSA) ؟ | Yes/No |
| Awareness | Source of health information about PCa | ما المصدر المأخوذة منه هذه المعلومات الصحية عن سرطان البروستات؟ | Multiple-response field; observed sources include healthcare provider; media; journals/internet; relatives/friends; “did not hear” |
| Knowledge | K1. Prostate cancer is the most common cancer in men. | مستوى المعرفة: [سرطان البروستات هو السرطان الأكثر شيوعًا عند الرجال] | Observed formats: Yes/No and Know/Don't know; scoring key=True for unambiguous Yes/No records |
| Knowledge | K2. I should be able to tell immediately if I have a prostate problem. | مستوى المعرفة: [يجب أن أكون قادراً على معرفة إذا كنت أعاني من مشكلة في البروستات فوراً] | Observed formats: Yes/No and Know/Don't know; scoring key=False for unambiguous Yes/No records |
| Knowledge | K3. Black men are more likely to develop and die from prostate cancer than other men. | مستوى المعرفة: [الرجال ذوو البشرة السوداء أكثر عرضة للإصابة والوفاة بسرطان البروستات مقارنة بغيرهم من الرجال] | Observed formats: Yes/No and Know/Don't know; scoring key=True for unambiguous Yes/No records |
| Knowledge | K4. Having a family member with prostate cancer increases the likelihood of developing prostate cancer. | مستوى المعرفة: [وجود شخص في العائلة مصاب بسرطان البروستات يزيد من احتمال الإصابة بسرطان البروستات] | Observed formats: Yes/No and Know/Don't know; scoring key=True for unambiguous Yes/No records |
| Knowledge | K5. Frequent nighttime urination may be a sign of prostate cancer. | مستوى المعرفة: [الاستيقاظ المتكرر في الليل للتبول قد يكون علامة على سرطان البروستات] | Observed formats: Yes/No and Know/Don't know; scoring key=True for unambiguous Yes/No records |
| Knowledge | K6. A diet high in fat will reduce the likelihood of developing prostate cancer. | مستوى المعرفة: [النظام الغذائي الغني بالدهون سيُقلل من احتمال الإصابة بسرطان البروستات] | Observed formats: Yes/No and Know/Don't know; scoring key=False for unambiguous Yes/No records |
| Knowledge | K7. The two main tests for detecting prostate cancer are the PSA blood test and digital rectal examination. | مستوى المعرفة: [الاختباران الأساسيان للكشف عن سرطان البروستات هما فحص الدم المعروف باسم المستضد النوعي للبروستات والفحص الشرجي الرقمي، حيث يدخل الإصبع المغلف بالقفاز داخل المستقيم لتحسس البروستات] | Observed formats: Yes/No and Know/Don't know; scoring key=True for unambiguous Yes/No records |
| Knowledge | K8. Conducting only one of the two tests (PSA or DRE) is sufficient to detect prostate cancer. | مستوى المعرفة: [إجراء أحد الفحصين فقط، فحص المستضد النوعي للبروستات أو الفحص الشرجي الرقمي، يكفي للكشف عن سرطان البروستات] | Observed formats: Yes/No and Know/Don't know; scoring key=False for unambiguous Yes/No records |
| Knowledge | K9. Early screening for prostate cancer cannot determine whether a person has prostate cancer. | مستوى المعرفة: [الفحص المبكر لسرطان البروستات لا يمكنه تحديد ما إذا كان الشخص مصابًا بسرطان البروستات] | Observed formats: Yes/No and Know/Don't know; scoring key=False for unambiguous Yes/No records |
| Knowledge | K10. It is often suggested that Black men over age 40 undergo prostate cancer screening annually. | مستوى المعرفة: [يُقترح غالبًا أن يقوم الرجال ذوو البشرة السوداء فوق سن الأربعين بإجراء فحص سرطان البروستات سنويًا] | Observed formats: Yes/No and Know/Don't know; scoring key=True for unambiguous Yes/No records |
| Knowledge | K11. Men aged 50 years and younger are at high risk of developing prostate cancer. | مستوى المعرفة: [الرجال الذين تبلغ أعمارهم 50 عاماً فأقل هم في خطر مرتفع للإصابة بسرطان البروستات] | Observed formats: Yes/No and Know/Don't know; scoring key=False for unambiguous Yes/No records |
| Knowledge | K12. A family history of prostate cancer is a risk factor. | مستوى المعرفة: [التاريخ العائلي لسرطان البروستات يُعدّ عامل خطورة] | Observed formats: Yes/No and Know/Don't know; scoring key=True for unambiguous Yes/No records |
| Testing/practice | Ever undergone testing | هل خضعت للفحص؟ | Yes/No |
| Testing/practice | Undergone testing during past year | هل خضعت للفحص خلال السنة الماضية؟ | Yes/No |
| Testing/practice | Reasons for not undergoing testing | أسباب عدم إجراء الفحص: | Multiple responses allowed |
| Testing/practice | Reasons for undergoing testing | أسباب إجراء فحص: | Multiple responses allowed |
| Willingness | Willing to undergo testing | هل لديك الرغبة في إجراء فحص؟ | Yes/No |
| Willingness | Reasons for not being willing | أسباب عدم الرغبة في إجراء فحص: | Multiple responses allowed |
| Willingness | Reasons for being willing | أسباب الرغبة في إجراء فحص: | Multiple responses allowed |

# Knowledge scoring protocol

Only records with 12 complete, unambiguous single Yes/No responses were scored (n=568). For each item, the response matching the source-derived answer key in Table 5 was coded 1 and the opposite single response 0. Records using Know/Don't know labels (n=393), the one mixed-format record, and Yes/No-labelled records containing at least one dual “Yes, No” selection (n=85) were not assigned a composite factual knowledge score. They remained in all other analyses.

Raw total: 0-12. Modified Bloom mapping used in the analysis: poor=0-7; fair=8-9; good=10; excellent=11-12; limited=0-9; adequate=10-12. KR-20 in the 568 scorable respondents was 0.235.

Important measurement note: The answer key is reproduced as the study's source-derived coding protocol. Some statements, particularly those about screening tests and age/race recommendations, reflect historical or context-dependent concepts and should not be interpreted as universal contemporary clinical guidelines. The shortened 12-item instrument was not independently validated in Palestine in the available study records.

# Observed barrier options among never-tested participants

| **Reason category** | **Selected n** | **% of never-tested (n=988)** |
| --- | --- | --- |
| Low perceived personal risk | 546 | 55.3% |
| No physician/healthcare-provider recommendation | 426 | 43.1% |
| No time | 124 | 12.6% |
| Fear of discovering prostate cancer | 39 | 3.9% |
| Perceived test as not useful | 32 | 3.2% |

Multiple responses were allowed. Fifty-seven of 988 never-tested participants had no recorded response in the barrier field.
